# Surgery

**Published:** 1877-12

**Authors:** 


### I. Surgery.

Amputation of the Penis; Transplantation of the Urethra. Wedemeyer. (*Archiv der Heilkunde XVIII.*, 6.)—The ordinary method of amputating the penis is always followed by the great inconvenience that the patient cannot urinate in the erect posture without wetting his clothes. Prof. Thiersch has successfully overcome this difficulty in the following case by transplanting the remnants of the urethra into the perinæum.

In November, 1874, a man 48 years old, greatly emaciated, was admitted into the hospital with an epithelial cancer of the penis. The whole organ, from the urethral orifice to the roof, was the seat of one large ulceration, surrounded by hard, raised and everted margins. The urethra was exposed to the extent of two centimetres; in the inguinal regions existed a number of hard, indolent, enlarged glands.

Under the antiseptic spray the penis was amputated at its root. The cut surface did not exhibit any morbid changes in the tissues. The profuse bleeding being arrested by catgut ligatures, the stump was secured by a strong catgut loop, and a catheter introduced into the urethra.

From the lower margin of the wound an incision was then carried along the scrotal raphé into the perinæum, thus dividing the scrotal sac into halves. By deepening this incision the surgeon could reach the urethra and dissect it off its surroundings towards the bladder to the length of two centimetres. This movable piece of the urethra was turned down to the perinæum and fastened to it by stitching the mucous lining of the urethra to the external integument. The whole wound was closed by catgut sutures; drainage tubes were put into both angles of the wound; a flexible catheter was kept in the transplanted urethra, and a salicylated dressing applied.

During the convalescence the patient had an attack of erysipelas, which caused his recovery to be rather slow. Still, in January, 1875, he had gained sufficient strength to undergo a second operation. A hard carcinomatous gland in the right inguinal fossa had to be extirpated. All the other indolent glandular tumors had disappeared by this time. In February he was discharged from the hospital. He could then, by raising the scrotum, discharge the urine at an angle of 45 degrees, in the erect posture, without soiling his clothes or his body. A re-examination, made a year later, showed the condition of the transplanted urethra and its satisfactory function still unchanged.

A PECULIAR EXTIRPATION OF A KIDNEY. Langenbuch. (*Berliner Klin. Wochenschrift*, 1877. N: 24.) The author describes a highly interesting case of the successful extirpation of a pathologically changed kidney, which was taken for a tumor.

A robust woman, aged 32 years, suffered during one and a half years from heavy pains in the region of the left kidney. Palpation could detect a solid and somewhat movable tumor of six to eight centimetres diameter, sensitive to pressure, apparently situated in or outside of the lumbar muscles. The overlying skin was entirely loose.

It was supposed to be a malignant tumor; not, however, originating in the kidney, as the urine was free from abnormal substances, and the tumor appearing to be located in the muscular tissue. The apex of the tumor reached into the upper muscular layer, to which it was found adherent by cicatricial tissue.

The finger, passing round the exposed tumor, detected a pedicle which was ligated, whereupon the tumor was cut off below the ligature. Suddenly the ligature slipped off; but the expected profuse bleeding did not appear. Only small veins were bleeding. An aperture which could have belonged to a carotid artery emitted no blood, and admitted bougies to the length of 15 centimetres. It was the ureter. The tumor was the kidney, which was changed into a cicatrized sac. The histological examination showed a complete degeneration of the tissues, whose original structure could no longer be ascertained. An inflammatory process, which had destroyed the glandular tissue, was considered the cause of the formation of the sac, but it is difficult to account for the abnormal position in which the kidney was found.

Malignant Osseous Tumor of Inferior Extremity of Femur.—M. LeDentu. (*L' Union Médicale*, Oct. 16, 1877.) LeDentu presented an osseous tumor of the inferior extremity of the femur, to the *Société de Chirurgie* Oct. 10, 1877.

It was the case of a woman of 50 years, of usual health until four and a half years since, when she was first taken with a tolerably sharp pain at the lower end of the femur on a level with the inner condyle; this pain was the only appreciable symptom felt by the patient until Feb., 1875, when a swelling appeared, which made rapid progress during the year 1876, and reached a considerable size in 1877. On entering the hospital Oct. 1st., under the care of M. LeDentu, the tumor measured 25 inches in circumference. The skin covering it was intact, but of a violet color and furrowed by varicose veins. Above the tumor the member was comparatively enlarged. Careful examination with the finger, revealed a deep tumefaction, indicating an osseous tumor. The diagnosis lay between an osteo-sarcoma and an enchondroma.

M. LeDentu at first thought of sarcoma, but finally, after having examined a plate representing a specimen of Dupuytren's, which presented a striking analogy with that of his patient, he changed his mind and regarded it as an enchondroma. It appeared to him that the tumor of his patient had the consistence and elasticity of an enchondroma. Besides, the general state of the patient was not bad; there was no sign of cachexia, except a little leanness which was habitual with the patient; finally, a careful exploration of the internal organs revealed no visceral lesion.

In consideration of these facts, which seemed to remove the idea of a cancerous tumor, M. LeDentu proposed disarticulation of the thigh, which was immediately accepted. The operation was performed by the two flap method, one anterior, the other posterior. Nothing unusual presented. The vessels were seized as cut by the hemostatic forceps, and the ligation of the femoral was reserved for the end of the operation. Toward the middle of the operation, however, the patient, who had been carefully chloroformed, presented symptoms of depression, from which she was brought by means of friction and flagellation.

The operation was then completed; the wound was united by means of three deep sutures and several superficial ones; four drainage tubes were inserted, and finally the dressing of Lister was used with all the minute precautions indicated by its author. Although M. LeDentu regards the case as very grave, yet he hopes, from all these conditions, that the patient may recover.

Examination of the specimen with the naked eye seems to confirm the diagnosis of M. LeDentu. The appreciable characters are those of enchondroma. The cartilaginous element seems to constitute the fundamental part of the tumor. The bone seems to have undergone profound alteration, even to the medullary canal, which is itself far from being healthy. M. LeDentu closed by saying that it was necessary to await microscopic examination, before pronouncing definitely upon the nature of the tumor.

The communication of M. LeDentu gave rise to an interesting discussion, in that it brought out the uncertainty which still exists on the subject of diagnosis and pathological anatomy of tumors, designated under the names osteo-sarcoma, enchondroma, cancer of the bones, etc.

M. Gillette regretted that M. Le Dentu had not dwelt at greater length upon the symptomatology of his patient, in particular on the character of the symptom, pain. In osteo-sarcoma, the spontaneous and the provoked pains are generally very sharp, and permit us to distinguish clinically this class of tumors from those designated as enchondromas, in which the pains are less severe. M. Gillette showed that the articular cartilages, in the specimen of M. LeDentu, remain intact.

M. Honel distinguishes two kinds of enchondroma, the benignant, which has no tendency to become general, and the malignant, which resembles cancer in its tendency to generalization; he thinks that the case of M. LeDentu belongs to the latter; the bone is as profoundly altered as possible, the alteration having reached the medullary canal already, the seat of intense osteo-myelitis. He does not believe that the patient of M. LeDentu can be saved.

M. Duplay thought it impossible to pronounce absolutely upon the diagnosis of the tumor, or the prognosis of the operation, after a simple examination of the specimen with the naked eye. This extremely complex specimen has the characters of sarcoma, of myxoma, and of enchondroma. Whatever it may be, M. Duplay believes it susceptible of early recurrence.

M. Déspres declares it a cancer, and bases his diagnosis upon the age of the patient, the date of the disease, four years, and finally on the general loss of flesh noted by M. LeDentu.

M. Marc Sèe has had occasion to observe at the *Maison Municipale de Santé* a little girl, who had a tumor situated at the upper extremity of the humerus, very close to the scapulo-humeral articulation. The rapid development of the tumor, which had distended, thinned, and inflamed the skin, obliged M. Sèe to disarticulate the member. The results of the operation were at first favorable, but cicatrization was not yet complete when several other tumors developed upon various portions of the head; the affection had become general.

M. Lucas-Championnière had seen a patient treated by M. Guyon with an enchondroma of the thigh, by disarticulation of the member. The patient got well of the operation, but died several months after, of cancer of the lung.

M. Amédée Forget said that under whatever name the tumor was designated, for diagnosis and prognosis, regard should be had, above all, to the clinical characters. For his part, the rapid development of the tumor would have made him fear a malignant tumor, and he would have abstained from interference. It is well to operate, but better still to abstain, when the operation must be fatally followed by recurrence. He asks if there is not in the present state of science, clinical characters indicating to the surgeon whether or not to operate. This point seems to him more important than the determination of the histological differences between tumors, a determination generally sterile in a practical point of view.

M. Nicaise saw a patient, in his service at the temporary hospital, with sarcoma of the leg, who had not experienced any morbid phenomena, except very sharp pain, which came on suddenly while walking, obliging him to stop and be carried home. The tumor appeared at once, and grew rapidly, acquiring in some weeks the large size it had when he entered the hospital. M. Nicaise amputated the leg, but the tumor recurred in the same place before the wound healed. The patient died of a recurrence in the lung.

M. Duplay had also seen a case of sarcoma of the calcaneum, long taken for an osteitis. He amputated, but in less than a year it recurred in the soft parts of the stump.

M. Marjolin thinks we should not hasten to conclude, after these recitals of recurrences after operation, that it is always best to abstain from operating in osseous tumors. We might thus allow patients to die that an operation might save. He remembers an operation he performed in the middle portion of the thigh of a *réligieuse* suffering from an osseous tumor, which distinguished surgeons of the hospitals, Michon, Herneyde Chégoin, and Désormeaux, had judged to be of a malignant nature. Now the operation was performed twenty years ago, and the patient still lives.

M. LeDentu, in answer to the various observations of which his communication had been the object, said that the pain did not offer any character sufficient to distinguish sarcoma from enchondroma, in spite of the affirmation of M. Gillette; as to the general state of the patient, he repeated that he had observed no sign of cachexia; the emaciation which she presented was normal, and belonged to the constitution rather than to the malady; if there had been cachexia he would naturally have abstained from operating. He recognized the gravity of the case. The rapidity of the development obliged him to consider it as malignant. But is that a reason for believing the case incurable, and to leave the patient to die rather than interfere? He did not think so. Science has no certain sign, either histological or clinical, which permits the distinguishing curable osseous tumors from those which are not so. In cases of doubt we should not abstain from operating, lest we allow patients to die, as M. Marjolin has said, which an operation might have saved. M. Marjolin is not the only surgeon who has seen patients get well after operations on osseous tumors regarded as malignant. Such examples are not rare, and he knew of several. In cases of doubt, then, we should act; sometime science may be able to tell in what cases it is best to abstain from operating.

L. W. C.

Ingrowing Toe-Nail,—Dr. A. H. Agard, of Oakland, Cal., contributes to the *Trans. Medical Society, California*, for 1876–7, an interesting paper on this frequently suggested and poorly understood subject, much neglected in the textbooks. Neither Fergusson nor Hamilton allude to it; Druitt and Erichsen each give it a paragraph; Holmes, in his *Treatise on Surgery*, gives it twenty lines; Gosselin, in his *Clinical Lectures on Surgery*, devotes nine pages to it; Gross, one.

The toe-nail grows from a matrix which lies in a fold of the skin near its base. In a large majority of cases it comes forward with a thickened recurved margin at either side, which lies in a groove in the soft parts that run from the matrix to the end of the digit. Thick nails are often provided with re-curved margins embedded in deep sulci on the sides of the digits. Dr. A. believes this conformation predisposes to the disease; also that it is often hereditary.

Anything may be an exciting cause that forces the incurved margin to impinge upon the skin lining the bottom of the groove, so as to bruise or irritate the parts. The most fruitful causes are wedging the foot into a narrow, funnel-shaped, stocking, wearing loose boots with high heels, cuneiform shaped toe, or boots too narrow for the foot.

The sulcus on the outer side of the great toe suffers oftenest; the lateral pressure operates first upon the inner and anterior angle of the nail, and the effect is to push the nail outward and to sink the margin deeper into its groove. With the increasing pressure, there is set up in the cuticle an effort to protect the true skin beneath. Epidermic cells are thrown out and left to harden into a protective covering. These, as they are cast off, are held in place by the margin of the nail until they form a hard pad under it, which becomes an offensive mass, having much of the tenderness that belongs to a true corn. The lateral pressure also forces the second upon the margin of the great toe, folding the inner border of the matrix under. This condition becoming permanent, (it is in youth that the disease is contracted) malformation of the nail with true inversion of the border results.

A curious and important point in this connection, is this: In walking, just at the last of each step, the weight of the body is thrown upon the end of the great toe. Especially is this true of the Caucasian who "toes out," but the Indian "toes in" by throwing himself forward in a loping gait, with a spring from all the toes. Place the femurs of an Indian and white man upon the table, resting upon their condyles at their lower end and upon their shafts at the other, the white man's femur will be found to rise but little, while the Indian's gives a very obtuse angle with the surface of the table. Place the head of each bone in its acetabulum, attach the foot and leg, and it can at once be seen what causes the difference in the gait. Not only the weight, but the last act in completing the step, forces the flexor muscles of this toe into action. But when the lateral expansion of the soft parts is denied, it finds room in the direction of the least resistance, which is at the front of the nail. The nail groove is converted finally into a pocket or pouch, into which the outer angle of the nail is pressed.

One predisposed to this disease, suffers first by a simple itching or tingling, not unlike that of a corn; occasionally severe twinges of pain are felt. Swelling and heat are then discovered, any slight injury causes an ulcer, and intense pain ensues.

In the *first* stage of this trouble, cut the nail short, trim both corners well back, keep the front rasped thin, and avoid exciting causes. If ulceration has taken place, relieve the bottom of the groove and the toe-nail pocket from pressure.

Two plans of treatment are proposed; the one temporizing, the other radical; for the former, remove the toe-nail, which gives relief, till a new one develops; or trim the free border of the nail back to the base, relieving the pressure, but take care to leave no spicula or sharp edges. This operation Dr. A. prefers to the former method, but it must be repeated every few months.

Three methods for radical cure deserve mention.

1. Removal of entire nail and matrix. The objection to this is that it leaves the top of the toe unprotected.

2. Removal of merely the margin of the nail with its matrix. The nail will remain narrower, and the recurring margin never be renewed.

3. Removal of soft parts along the border of the toe, taking away the groove and obstructing the growth of the angle of nail. This least objectionable method is as effectual a cure as removing the nail, but care should be taken to effectually ablate the nail growth and remove the upthrust of indurated tissue anterior to the outer angle. When the deformed matrix sends forward a malformed margin, remove both margin and matrix. In rare cases it may be proper to combine two operations in removal of margin of nail and side toe.

General anæsthesia is unnecessary; local application of snow, ice, or freezing mixture, is sufficient. When engorgement of tissue is extreme, the hemorrhage may be great, but no harm can follow.

Dr. A. dresses the wound with salicylic acid in substance, using the dry powders; and orders the dressings to be kept wet for a few hours with cold water. Profuse granulations should be repressed, but they do not come up under the use of salicylic acid. M. E. K.

Instrumental Dilatation of the Rectum for Operative Purposes. W. Hack. *Archiv. fur Klin. Chirurgie.* 1877. Bd. XXI. Hft. 2.—The operation is performed, after the rectum is carefully cleansed, in profound chloroform narcosis. The patients are placed upon the table on their backs, with pelvis elevated, the thighs strongly flexed upon the abdomen. With the well-oiled speculum (Simon used them from 5 to 5½ ctm. in diameter, and from 15 to 17 ctm. in circumference), the rear wall of the rectum is pushed aside; the side walls are held back by a vaginal tenaculum, and the projecting skin of the anus by double hooks, or by a flat speculum. If necessary the sphincter is cut backwards in the raphé, as such incision into the lower end of the rectum facilitates the access to the cavity of the os sacrum. In cases of recto-vaginal fistula, the edges of the fistula are now pared off, if possible transversely; the sutures are partially put in by the means of Simon's needle-holder, and fine Chinese silk; or with Langenbeck's needles, used for staphylorrhaphy.

In the after treatment a strong cathartic was administered by Simon, at least every second day, the method of constipation having proved dangerous. The removal of the sutures is effected through the vagina; the slight formation of pus around the threads had always enlarged the canals of the stitches sufficiently to admit of the passage of the knots. The cases operated upon by Simon present the best proof of the practicability of his method; we can here only speak of them in a few words. The first case was a person with two small vesico-vaginal fistulæ, and a large recto-vaginal fistula. The fistula was unsuccessfully operated upon through the vagina, whereas the cure was effected by two operations from the rectum; the vesico-vaginal fistulæ were also cured. In a second case, a woman 45 years of age, was cured of a recto-vaginal fistula, which was immovable, and situated 1 ctm. from the orifice of the uterus.

The third case occurred in a woman, 32 years old, who had contracted a recto-vaginal fistula during an attack of typhus, the tube of the injection syringe having perforated an ulcerated place of the rectum. The fistula was cured after an operation through the rectum, only leaving small places of the size of a pin's head, which afterwards healed up under cauterization.

The fourth case is that of a soldier, who had contracted two fistulæ by a gun-shot wound through the bladder and rectum.

The fifth case gives the best proof of the practicability of this method. The patient, 22 years old, suffered from a vesico-vaginal fistula, besides there existed a communication between the bladder and the rectum, and an occlusion of the vagina.

Fat-Embolism Resulting from Rupture of a Fatty Liver.—Hamilton. (*British Med. Journal*, Oct. 6, 1877.) Fat-embolism is a pathological lesion which has been described within the past few years more especially by Zenker, Wagner and Busch. It generally happens that the subject of it has sustained a fracture of bone, or some operation has been performed necessitating the opening of the medullary cavity of a bone. The oil which so escapes into the surrounding parts becomes absorbed by the neighboring blood-vessels and is carried to the right side of the heart, and subsequently to the lungs. Here it gives rise to the most complete capillary embolism, entirely precluding the passage of the blood through the organ, the patient accordingly dying from gradual carbonic acid poisoning. These cases of death in a few hours after a simple fracture of bone, or operation on bone, were, up to the time that this important discovery was made, totally unaccounted for; the patient was generally said to have died from "shock." In the case here reported the oil, which acted as embolon, was derived from an unusual source. The patient was a lad about fourteen years old, and a sailor. While aloft he slipped and fell a considerable distance onto the ship's deck, alighting on his right side. Although stunned, he soon recovered sufficiently to walk about the deck. In an hour or two afterwards, however, he became very much distressed; his breathing became embarassed, coma ensued, and he died a few hours after the injury. At the post-mortem examination, the liver was found to have sustained a few small ruptures, from which a little blood had flowed, but only a comparatively small quantity. The liver itself, however, was *peculiarly fatty*, and, for a lad of his age, unusually so; whereas the other organs seemed perfectly normal, so that the lungs and kidneys were retained as specimens of healthy organs, and were hardened in chromic acid for microscopic examination.

In due time, sections were made of the lung and kidney, and in order to bring out their outlines more prominently, a half per cent. solution of perosmic acid was employed as a staining re-agent. The most extraordinary appearance was revealed by this means in the lung. The whole of the middle and smallest sized arteries were found to be plugged with oil, which now had a black color, owing to the action of the perosmic acid. Even the minutest capillaries were completely choked up with oil. The oil was generally in one large elongated mass, occupying the lumen of the vessel for a considerable distance, or it was in drops of various sizes closely aggregated together. Similar embolisms were detected in the kidneys, but only in small number.

